# Intravenous immunoglobulin for chronic residual peripheral neuropathy in eosinophilic granulomatosis with polyangiitis (Churg–Strauss syndrome): a multicenter, double-blind trial

**DOI:** 10.1007/s00415-014-7618-y

**Published:** 2015-01-11

**Authors:** Haruki Koike, Kazuo Akiyama, Toyokazu Saito, Gen Sobue, The Research Group for IVIg for EGPA/CSS in Japan

**Affiliations:** 1 Department of Neurology, Nagoya University Graduate School of Medicine, Nagoya, 466-8550 Japan; 2 Clinical Research Center for Allergy and Rheumatology, National Hospital Organization Sagamihara National Hospital, Sagamihara, Japan; 3 Kitasato University School of Allied Health Sciences, Sagamihara, Japan

**Keywords:** Churg–Strauss syndrome, Eosinophilic granulomatosis with polyangiitis, Intravenous immunoglobulin, Neuropathy, Treatment

## Abstract

Eosinophilic granulomatosis with polyangiitis (EGPA), previously called Churg–Strauss syndrome, frequently affects the peripheral nervous system. We conducted a multicenter, double-blind, three-arm treatment period, randomized, pre-post trial to assess the efficacy of intravenous immunoglobulin (IVIg) administration for residual peripheral neuropathy in patients with EGPA that is in remission, indicated by laboratory indices. Twenty-three patients were randomly assigned into three groups, in which the timing of IVIg and placebo administration was different. Each group received one course of intervention and two courses of placebo at 2-week intervals. Treatment effects were assessed every 2 weeks for 8 weeks. The primary outcome measure, the amount of change in the manual muscle testing sum score 2 weeks after IVIg administration, significantly increased (*p* = 0.002). The results over time suggested that this effect continued until the last assessment was done 8 weeks later. The number of muscles with manual muscle testing scores of three or less (*p* = 0.004) and the neuropathic pain scores represented by the visual analogue scale (*p* = 0.005) also improved significantly 2 weeks after IVIg administration. This study indicates that IVIg treatment for EGPA patients with residual peripheral neuropathy should be considered even when laboratory indices suggest remission of the disease.

## Introduction

Peripheral neuropathy is caused by primary and secondary vasculitides of various etiologies [1, 2]. Eosinophilic granulomatosis with polyangiitis (EGPA), previously called as Churg–Strauss syndrome, frequently involves the peripheral nervous system [3–5]. As opposed to involvement of the heart, lungs, gastrointestinal tract, kidneys, or central nervous system, which can be life threatening, peripheral neuropathy caused by vasculitis alone does not significantly affect patient survival [2, 6]. However, peripheral neuropathy can significantly disrupt day-to-day functioning and quality of life of patients because of weakness or pain in the extremities [7, 8]. Administration of corticosteroids alone or a combination of corticosteroids and immunosuppressive agents such as cyclophosphamide typically achieves remission and results in good survival rates in patients with EGPA [9–11]; however, patients may suffer from chronic residual functional deficits caused by neuropathy even after remission has been achieved [8, 12, 13]. Although such deficits may disrupt the day-to-day functioning and quality of life of patients, studies to target the residual neuropathy are still scarce.

In this article, we assessed the efficacy of intravenous immunoglobulin (IVIg) for EGPA from the viewpoint of residual neuropathy during disease remission indicated by laboratory indices.

## Methods

### Patients

**Fig. 1:**
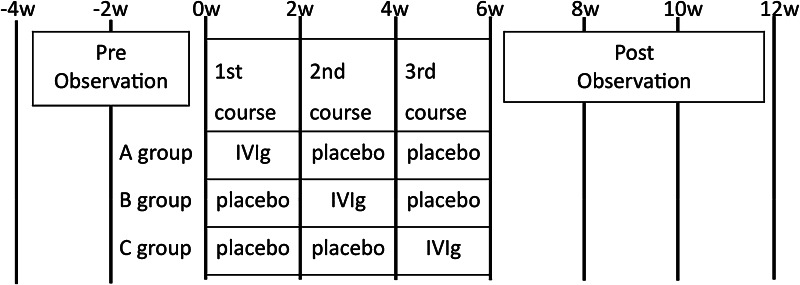
Treatment and observation schedule. Patients were randomly assigned into three groups (groups *A*, *B*, and *C*), in which the timing of intravenous immunoglobulin (IVIg) and placebo administration was different. Each group received one course of IVIg and two courses of placebo at 2-week intervals. IVIg was administered during the first course for group *A*, the second course for group *B*, and the third course for group *C*. Medical examinations were done on a 2-week interval schedule starting from 4 weeks before the first infusion to 8 weeks after the start of the third course

A multicenter, double-blind, 3-arm treatment period, randomized, pre-post trial was done at 23 hospitals (Fig. 1). As this disease is relatively rare, we adopted this design rather than a placebo-controlled, parallel-group trial. Patients who fulfilled the following criteria were included in the study: (1) a diagnosis of EGPA [14–16]; (2) aged between 20 and 74 years; (3) manifestation of weakness of both less than three with manual muscle testing (MMT) [17] in more than one muscle and MMT sum score of less than 130 as described below; and (4) disease remission induced by more than 4 weeks of steroid treatment. Steroid treatment was defined as the use of more than 40 mg/day of prednisolone (or an equivalent dosage of another steroid) in the initial phase, followed by a reduction of dosage and a subsequent maintenance therapy of 5–20 mg/day of prednisolone (or an equivalent dosage of another steroid) for more than 4 weeks. A diagnosis of EGPA was based on the 2012 revised International Chapel Hill Consensus Conference Nomenclature and the diagnostic criteria of the Ministry of Health and Welfare of Japan for definite EGPA (1998) [14–16]. All patients had asthma, eosinophilia, and multiple mononeuropathy. Patients also showed histological findings consistent with EGPA and/or a characteristic clinical course [14, 15].

MMT was assessed at 30 points for each patient by neurologists, and scored as: 5, normal; 4, good; 3, fair; 2, poor; 1, trace; and 0, zero [17]. The following movements (and their associated muscles) were examined bilaterally: elbow flexion at antebrachial supination (biceps brachii), elbow extension (triceps brachii), elbow flexion at antebrachial mid-position (brachioradialis), wrist flexion (flexor carpi radialis and flexor carpi ulnaris), wrist extension (extensor carpi radialis longus, extensor carpi radialis brevis, and extensor carpi ulnaris), extension of metacarpophalangeal joint of thumb (extensor pollicis longus and extensor pollicis brevis), opposition of thumb (opponens pollicis), abduction of fingers (interossei dorsales), and adduction of fingers (interossei palmares) in the upper extremities, and knee flexion (hamstring), knee extension (quadriceps), ankle plantar flexion (gastrocnemius and soleus), ankle inversion (tibialis posterior), ankle dorsiflexion (tibialis anterior), and ankle eversion (peroneus longus and peroneus brevis) in the lower limbs. Because the strengths of muscles that cause extension of the metacarpophalangeal joint of the thumb, abduction of the fingers, and adduction of the fingers were fine, we set the highest scores of these points to four to increase the reproducibility of the results of the assessment. Therefore, the full score of the sum of MMTs was 144.

Exclusion criteria were: history of allergy or shock to IVIg; IgA deficiency; severe renal failure defined as serum blood urea nitrogen levels no less than 40 mg/dl or serum creatinine levels no less than 4 mg/dl; history of cerebrovascular or cardiovascular disease; high risk of thrombosis; hemolytic or hemorrhagic anemia; congestive heart failure of NYHA functional class III or worse; immunodeficiency inappropriate to enroll in this study; severe muscle weakness; pregnancy or breast feeding; previous participation in a Phase II trial; improvement of more than 10 % on MMT sum score during 4-week pretreatment period; participation in other clinical trials within the 12 weeks prior to giving informed consent; and ineligibility for enrollment judged by physicians-in-charge.

This study is registered in the JAPIC clinical trials registry, number JapicCTI-060242(ja)(en). Patients provided written informed consent before enrolment. The protocol was approved by the institutional review board at each participating center. The study was done in accordance with the Declaration of Helsinki and good clinical practice.

### Randomization and masking

Patients were randomly assigned into three groups (groups A, B, and C), in which the timing of IVIg (freeze-dried sulfonated human normal immunoglobulin, 0.4 g/kg for five consecutive days) and placebo (0.9 % isotonic sodium chloride solution) administration was different. Each group received one course of IVIg and two courses of placebo at 2-week intervals. IVIg was administered during the first course for group A, the second course for group B, and the third course for group C. This 3-arm design better ensures blindness compared to a 2-arm design. Patients were assigned to a computer-generated randomization list.

Patients and investigators were masked to treatment allocation. Vials and drip chambers were covered and opaque routes were adopted for both IVIg and placebo to maintain blindness of the study drug for patients and investigators. Surgical tape was put on the drip chambers so that the liquid surface was not visible. A trusted third party verified the blindness. In addition, preparation and cleanup of the drugs were done by a person in charge who was unrelated to the patients or the investigators.

The drug codes were broken and made available for data analysis when the study was completed and the data files had been verified.

### Procedures

The same volume and infusion rate were used for IVIg and placebo administration. Medical examinations were done on a 2-week interval schedule starting from 4 weeks before the first infusion to 8 weeks after the start of the third course. The medical examination was performed immediately before the infusion, when they overlapped. Blood and urine samples were obtained for the screening of general conditions at 4 weeks prior to the experiment, immediately before the first infusion, and at 2-week intervals thereafter, until 8 weeks after the start of the third course. Consecutive neurological examinations were performed by one neurologist for each patient during the study.

The primary outcome measure was the amount of change in the MMT sum score 2 weeks after IVIg administration. The secondary measures were changes in the number of muscles with an MMT score of 3 or less, visual analogue scale (VAS) scores [18], and a modified Barthel index [19] two weeks after IVIg administration. Changes in vibratory sensation in the distal portions of the lower limbs and deep tendon reflexes also were assessed. Nerve conduction studies were performed using standard methods. Hematological examinations were assessed by the items shown in Table 1. Urinary protein and sugar were also assessed. These indices were assessed before IVIg/placebo and every 2 weeks thereafter. Electrocardiography was performed at baseline and at 6 and 12 weeks after the start of the first course. The results of total protein and IgG levels that may increase after IVIg administration were blinded for examiners.

**Table 1:** Background of patients

Parameters	Group A (*n* = 8)	Group B (*n* = 8)	Group C (*n* = 7)	All patients (*n* = 23)	*p* values
Age	50.3 ± 17.2	61.0 ± 16.2	62.6 ± 4.5	57.7 ± 14.7	0.203
Sex (M:F)	3:5	2:6	2:5	7:16	1.000
Body weight (kg)	60.61 ± 13.58	49.80 ± 9.08	60.04 ± 9.93	56.68 ± 11.76	0.120
Duration of disease (years)	1.66 ± 1.55	0.81 ± 0.60	5.23 ± 10.78	2.45 ± 6.02	0.344
MMT sum score	110.00 ± 12.56	113.19 ± 12.57	106.29 ± 21.36	109.98 ± 15.26	0.702
Modified Barthel index	87.1 ± 15.2	79.5 ± 27.4	78.1 ± 7.3	81.7 ± 18.5	0.612
Visual analogue scale	64.75 ± 32.19	80.75 ± 20.25	61.86 ± 28.84	69.43 ± 27.56	0.365
Laboratory findings					
White blood cell (no./mm^3^)	8,275 ± 2,468	6,888 ± 1,063	8,186 ± 1,981	7,765 ± 1,949	0.300
Eosinophil (no./mm^3^)	55.0 ± 45.0	127.3 ± 98.0	251.5 ± 331.7	140.0 ± 200.9	0.165
Hemoglobin (g/dl)	14.00 ± 1.94	12.61 ± 1.61	13.96 ± 1.85	13.50 ± 1.84	0.246
Platelet (×10,000 no./mm^3^)	26.08 ± 4.76	22.29 ± 3.10	22.94 ± 3.01	23.80 ± 3.96	0.125
Aspartate transaminase (IU/L)	16.1 ± 3.1	18.8 ± 7.8	19.6 ± 3.2	18.1 ± 5.3	0.426
Alanine transaminase (IU/L)	17.9 ± 9.3	24.4 ± 30.3	21.7 ± 7.9	21.3 ± 18.6	0.796
Lactate dehydrogenase (IU/L)	208.9 ± 53.7	204.0 ± 38.3	230.1 ± 28.3	213.7 ± 41.6	0.461
Blood urea nitrogen (mg/dL)	12.03 ± 4.33	14.58 ± 4.37	15.33 ± 5.61	13.92 ± 4.76	0.380
Creatinine (mg/dL)	0.613 ± 0.163	0.585 ± 0.104	0.733 ± 0.202	0.640 ± 0.165	0.194
C-reactive protein (mg/dL)	0.051 ± 0.055	0.110 ± 0.092	0.094 ± 0.105	0.085 ± 0.086	0.385
Thrombomodulin (FU/mL)	2.86 ± 0.69	3.09 ± 0.67	3.46 ± 0.55	3.12 ± 0.66	0.222
IgG (mg/dL)	817.1 ± 191.8	892.9 ± 200.1	840.9 ± 343.5	850.7 ± 240.2	0.827
IgE (IU/mL)	202.88 ± 109.52	254.98 ± 335.70	113.66 ± 128.27	193.84 ± 218.17	0.473
MPO-ANCA^a^					
Positive/negative^b^	4/4	0/8	2/5	6/17	0.069
Daily amount of prednisolone at entry (mg/day)	15.75 ± 5.53	13.44 ± 6.40	14.00 ± 5.84	14.41 ± 5.75	0.724
Use of immunosuppressants	2 cases (25.0 %)	3 cases (37.5 %)	2 cases (28.6 %)	7 cases (30.4 %)	1.000

*MMT* manual muscle testing, *MPO*-*ANCA* myeloperoxidase-antineutrophil cytoplasmic antibodies

^a^Assessed at the time of diagnosis. MPO-ANCA was negative in all cases at the time of entry

^b^Negative value was defined as less than 10 EU

### Statistical analysis

Quantitative data were presented as the mean ± standard deviation (SD). To assess the demographic features of patients, the *χ* ^2^ test, Fisher’s exact test, and one-way analysis of variance (ANOVA) were used as appropriate. The primary and secondary endpoints for change after IVIg administration were assessed by the use of a paired *t* test. For comparison of the secondary endpoints with placebo, we used a two-sample *t* test and a one-way ANOVA to calculate the differences between the groups. Two-sided *p* values less than 0.05 were deemed statistically significant. Statistical analyses were done using SAS (version 9.1.3).

## Results

A total of 23 patients were assigned into groups A to C (eight patients for group A, eight patients for group B, and seven patients for group C). All patients were assessed as an analysis object. The demographics and characteristics assessed before IVIg/placebo administration were not significantly different among the three groups (Table 1). Disease activity indicated by eosinophil count, C-reactive protein, IgE, and myeloperoxidase-antineutrophil cytoplasmic antibodies (ANCA) were within the normal range, except for a slight elevation of IgE levels in five, three, and two patients of the groups A, B, and C, respectively. The findings from electrocardiography were unremarkable in all patients. In addition to muscle weakness, all patients manifested sensory deficits. The presence of neuropathy was confirmed by a reduction or absence of deep tendon reflexes in the affected limbs and/or abnormalities in nerve conduction indices. Pathological reflexes were absent in all patients.

Sequential changes in clinical scores are summarized in Tables 2 and 3. The primary outcome measure, the amount of change in the MMT sum score 2 weeks after IVIg administration, was 7.13 ± 9.76 (*p* = 0.002), which was a significant increase compared to the baseline score (113.37 ± 16.02–120.50 ± 11.91) (Table 2). Significantly increased scores (*p* < 0.001), compared to pretreatment scores, were also observed 4 weeks (124.83 ± 10.97), 6 weeks (125.37 ± 10.23), and 8 weeks (127.02 ± 9.34) later.

**Table 2:** Sequential changes in MMT sum scores after intravenous immunoglobulin administration

		a. Baseline^a^	b. 2 weeks	c. 4 weeks	d. 6 weeks	e. 8 weeks	*p* values*			
							b–a	c–a	d–a	e–a
MMT sum scores and the change in MMT sum scores										
Group A (*n* = 8)		110.00 ± 12.56	118.13 ± 11.15	122.75 ± 9.99	123.81 ± 10.16	124.50 ± 9.07	0.046	0.015	0.016	0.011
		–	8.13 ± 9.49	12.75 ± 11.29	13.81 ± 12.44	14.50 ± 11.87				
Group B (*n* = 8)		117.19 ± 11.50	123.00 ± 13.20	128.06 ± 13.05	127.81 ± 11.40	128.63 ± 11.21	0.022	0.019	0.014	0.006
		–	5.81 ± 5.62	10.88 ± 10.12	10.63 ± 9.24	11.44 ± 8.36				
Group C (*n* = 7)		112.86 ± 23.83	120.36 ± 12.49	123.50 ± 10.22	124.36 ± 9.99	128.07 ± 8.01	0.214	0.151	0.125	0.059
		–	7.50 ± 14.26	10.64 ± 17.12	11.50 ± 17.09	15.21 ± 17.29				
All patients (*n* = 23)		113.37 ± 16.02	120.50 ± 11.91	124.83 ± 10.97	125.37 ± 10.23	127.02 ± 9.34	0.002	<0.001	<0.001	<0.001
		–	7.13 ± 9.76^b^	11.46 ± 12.41	12.00 ± 12.57	13.65 ± 12.30				

*MMT* manual muscle testing

* Paired *t* test for change from baseline

^a^Baseline scores were those immediately before intravenous immunoglobulin administration

^b^Primary endpoint

**Table 3:** Sequential changes in secondary clinical scores after intravenous immunoglobulin administration

	a. Baseline^a^	b. 2 weeks	c. 4 weeks	d. 6 weeks	e. 8 weeks	*p* values*			
						b–a	c–a	d–a	e–a
Number of muscles with MMT scores of three or less									
Group A (*n* = 8)	11.0 ± 6.1	7.0 ± 4.4	5.5 ± 4.3	5.3 ± 3.8	5.1 ± 3.3				
Group B (*n* = 8)	7.8 ± 4.8	6.0 ± 4.6	4.1 ± 4.0	4.3 ± 3.8	4.3 ± 3.8				
Group C (*n* = 7)	10.9 ± 9.4	8.7 ± 6.2	7.9 ± 5.2	7.4 ± 5.3	5.3 ± 3.2				
All patients (*n* = 23)	9.8 ± 6.8	7.2 ± 5.0	5.7 ± 4.6	5.6 ± 4.3	4.9 ± 3.3	0.004	0.002	0.001	<0.001
Visual analogue scale									
Group A (*n* = 8)	64.75 ± 32.19	59.75 ± 33.72	59.13 ± 30.43	57.00 ± 29.75	55.50 ± 33.55				
Group B (*n* = 8)	77.63 ± 21.49	71.13 ± 21.75	68.63 ± 26.28	70.00 ± 23.72	70.81 ± 24.28				
Group C (*n* = 7)	59.43 ± 31.41	56.29 ± 29.83	49.71 ± 34.44	53.29 ± 28.00	51.43 ± 28.26				
All patients (*n* = 23)	67.61 ± 28.40	62.65 ± 28.23	59.57 ± 29.98	60.39 ± 26.98	59.59 ± 28.92	0.005	0.002	0.004	0.021
Modified Barthel index									
Group A (*n* = 8)	87.1 ± 15.2	89.5 ± 11.2	92.1 ± 8.0	92.3 ± 7.9	93.4 ± 6.9				
Group B (*n* = 8)	80.0 ± 26.9	84.5 ± 25.3	85.1 ± 25.6	87.3 ± 21.4	87.4 ± 21.2				
Group C (*n* = 7)	85.9 ± 10.7	86.4 ± 10.8	91.1 ± 8.0	91.1 ± 8.0	91.9 ± 6.8				
All patients (*n* = 23)	84.3 ± 18.6	86.8 ± 16.7	89.4 ± 16.0	90.2 ± 13.7	90.8 ± 13.3	0.107	0.020	0.009	0.005

*MMT* manual muscle testing

* Paired *t* test for change from baseline

^a^Baseline scores were those immediately before intravenous immunoglobulin administration

As for the secondary measures, the change in the number of muscles with MMT scores of 3 or less 2 weeks after IVIg administration was −2.7 ± 4.0 (*p* = 0.004), which was a significant reduction compared to the baseline score (9.8 ± 6.8–7.2 ± 5.0) (Table 3). Significantly reduced scores, compared to pretreatment scores, were also observed at 4 weeks (5.7 ± 4.6, Δ (the amount of change from baseline) = −4.1 ± 5.6, *p* = 0.002), 6 weeks (5.6 ± 4.3, Δ = −4.3 ± 5.2, *p* = 0.001), and 8 weeks later (4.9 ± 3.3, Δ = −5.0 ± 5.6, *p* < 0.001). The amount of change in the VAS 2 weeks after IVIg administration was −4.96 ± 7.72 (*p* = 0.005), which was a significant reduction from the baseline score (67.61 ± 28.40–62.65 ± 28.23). Significantly reduced scores, compared to pretreatment scores, were also observed at 4 weeks (59.57 ± 29.98, Δ = −8.04 ± 10.93, *p* = 0.002), 6 weeks (60.39 ± 26.98, Δ = −7.22 ± 10.71, *p* = 0.004), and 8 weeks (59.59 ± 28.92, Δ = −8.02 ± 15.42, *p* = 0.021). On the contrary, the modified Barthel index was not significantly reduced (84.3 ± 18.6 for pretreatment and 86.8 ± 16.7, 89.4 ± 16.0, 90.2 ± 13.7, and 90.8 ± 13.3, respectively, for 2, 4, 6, and 8 weeks after treatment).

The effect of IVIg was compared to that of placebo during the first course of infusion (Table 4). Patients in group A (*n* = 8), who were administered IVIg, showed a significantly greater reduction in the number of muscles with MMT scores of three or less than those in groups B and C (*n* = 15), who received placebo (−4.0 ± 5.3 versus −0.5 ± 1.6, *p* = 0.028). Significant differences were not found between IVIg and placebo in the items of the indices of MMT sum score (8.13 ± 9.49 versus 3.13 ± 3.52), reduction of VAS (−5.00 ± 8.62 versus −5.13 ± 7.44), and increase of modified Barthel index (2.4 ± 4.3 versus 1.9 ± 5.7).

**Table 4:** Comparison between IVIg and placebo after the first course of administration

	Change from the baseline	Difference (95 % CI)	*p* values
MMT sum score			
IVIg (group A, *n* = 8)	8.13 ± 9.49	4.99 (−0.64, 10.63)	0.080
Placebo (groups B and C, *n* = 15)	3.13 ± 3.52		
Number of muscles with MMT scores of three or less			
IVIg (group A, *n* = 8)	−4.0 ± 5.3	−3.5 (−6.5, −0.4)	0.028
Placebo (groups B and C, *n* = 15)	−0.5 ± 1.6		
Visual analogue scale			
IVIg (group A, *n* = 8)	−5.00 ± 8.62	0.13 (−7.02, 7.28)	0.969
Placebo (groups B and C, *n* = 15)	−5.13 ± 7.44		
Modified Barthel index			
IVIg (group A, *n* = 8)	2.4 ± 4.3	0.5 (−4.3, 5.3)	0.827
Placebo (groups B and C, *n* = 15)	1.9 ± 5.7		

*MMT* manual muscle testing, *CI* confidence interval

Changes in the primary and secondary outcome measures after IVIg administration were not significantly associated with age, sex, body weight, duration of disease, or use of adjunctive immunosuppressants.

Adverse events—judged by the investigator—related to the study medication were reported in 14 patients (61 %). These included headache (4 events), elevation of alanine aminotransferase (3 events), lassitude (2 events), erythema (1 event), purpura (1 event), chest pain (1 event), itching at the site of infusion (1 event), swelling at the site of infusion (1 event), edema in the limbs (1 event), fever (1 event), elevation of aspartate aminotransferase (1 event), elevation of γ-glutamyl transpeptidase (1 event), elevation of lactate dehydrogenase (1 event), reduced platelet count (1 event), and reduced white blood cell count(1 event). All of them were mild or moderate and most of them spontaneously resolved. As for the serious adverse events, one patient suffered from pneumonia and another had a urinary tract infection. The former was considered to be related to the administration of steroids, and the latter appeared transiently 4 weeks after the administration of IVIg; therefore, these events were considered to be unrelated to IVIg administration.

## Discussion

Although a successful treatment strategy for the acute phase of EGPA has been established [9–11, 20], some patients show persistent disease activity in spite of treatment [9, 20]. In EGPA, cardiac, gastrointestinal, and renal involvement, especially the first one, may become the cause of death [3, 5, 21], and cardiac involvement significantly affects the mortality rate [5]. In a previous retrospective study of 28 patients with neuropathy due to EGPA who were diagnosed by sural nerve biopsy, the patients who responded well to the initial 4-week corticosteroid therapy regained self-controlled functional status in a long-term follow-up (determined by modified Rankin scale), whereas those that did not respond well to the initial corticosteroid therapy were more likely to lead a dependent existence [12]. Peripheral nerve involvement influences the ability of patients to perform the activities of daily living [8, 12]. That study indicated that residual neuropathy might exist even after laboratory indices had suggested that remission had been achieved. Therefore, a treatment strategy to improve residual neuropathy is needed even when the activity of the disease, as indicated by laboratory indices, is negative. Previous studies concerning treatments for vasculitides have mainly focused on improvements in laboratory indices or on survival rate. This is the first study to focus on improvements in functional deficits that remain in spite of laboratory indices indicating remission.

In this study, we elucidated the efficacy of IVIg for improving the residual neuropathy of patients with EGPA during disease remission indicated by laboratory indices after initial immunosuppressive treatment. The results of the MMT sum score as well as individual MMT scores of three or less over time suggested that the effects of IVIg continued during the 8-week follow-up period. As this disease is relatively rare, we adopted a study design to compare the amount of change in the MMT sum score before and after the treatment rather than a placebo-controlled, parallel-group trial. However, sub-analyses also revealed significantly better outcomes compared to placebo in the number of muscles with an MMT score of 3 or less. The scale of pain (i.e. VAS), which may significantly affect quality of life, was also significantly improved after treatment.

It has been suggested that IVIg treatment is effective for patients with small-to-medium vessel vasculitis, especially when disease activity persists after standard therapy [22]. As for EGPA, scores on the modified Rankin scale significantly improved after a combination therapy of plasmapheresis and IVIg in patients receiving both prednisone and cyclophosphamide [23]. However, unlike our patients, the patients included in that study still manifested abnormal laboratory indices including positive ANCA titers and C-reactive proteins at baseline [23].

The mechanisms by which IVIg improves the functional status of patients with EGPA have not yet been elucidated. The efficacy of IVIg for neuropathy has been reported in patients with Guillain–Barré syndrome [24], chronic inflammatory demyelinating polyneuropathy [25], painful neuropathy associated with Sjögren’s syndrome [26], and a subgroup of diabetic neuropathy [27]. A variety of mechanisms have been thought to be responsible for the effect of IVIg on neuropathy, including neutralization of autoantibodies, inhibition of complement pathways, alteration of Fc receptor expression, and alteration of cytokine profiles [28]. It is presumed that the specific action of IVIg varies depending on the underlying pathogenesis of a given disease. In EGPA, IVIg might control smoldering inflammation in the peripheral nervous system.

In our study, disease activity indicated by laboratory indices was negative in all patients. It is notable that the improvement of neurological deficits was observed even in patients considered classically as being in remission. Therefore, this study indicates that IVIg treatment for patients with residual neuropathy should be considered even when laboratory indices suggest remission of the disease. Differential clinical profiles between patients with and without ANCA have been reported [4]. Another study revealed that serum IgG4 levels are markedly elevated in active EGPA and correlate with the number of organ manifestations and disease activity [29]. These findings may suggest a participation of some peculiar mechanisms other than ANCA-related pathways in the pathogenesis of EGPA. Such unraveled mechanisms might induce smoldering disease activity, which could not be measured by conventional laboratory indices. Further studies are needed to elucidate the mechanisms underlying the efficacy of IVIg for EGPA.

## Appendix

Members of the Research Group for IVIg for EGPA/CSS in Japan are as follows: Naoki Hattori, Gen Sobue, Department of Neurology, Nagoya University Graduate School of Medicine; Masami Taniguchi, Kazuo Akiyama, Clinical Research Center for Allergy and Rheumatology, National Hospital Organization Sagamihara National Hospital; Toyokazu Saito, Kitasato University School of Allied Health Sciences; Hitoshi Kobayashi, Third Department of Internal Medicine, Iwate Medical University School of Medicine; Tamotsu Ishizuka, The First Department of Internal Medicine, Gunma University School of Medicine; Takeshi Fukuda, Department of Pulmonary Medicine and Clinical Immunology, Dokkyo Medical University School of Medicine; Seiji Minoda, Division of Rheumatology and Clinical Immunology, Jichi Medical University School of Medicine; Haruhito Sugiyama, Department of Respiratory Medicine, National Center for Global Health and Medicine; Yoshinari Takasaki, Department of Internal Medicine and Rheumatology, Juntendo University School of Medicine; Shigeko Inokuma, Department of Allergy and Immunological Diseases, Tokyo Metropolitan Komagome Hospital; Ken Ohta, Department of Internal Medicine, Teikyo University School of Medicine; Hirahito Endo, Department of Rheumatology and Infectious Diseases, Kitasato University School of Medicine; Takao Sugiyama, Department of Rheumatology, National Hospital Organization Shimoshizu National Hospital; Kyoichi Nomura, Department of Neurology, Saitama Medical Center; Makoto Nagata, Department of Respiratory Medicine, Saitama Medical University; Hiroshi Hayakawa, Department of Internal Medicine, National Hospital Organization Tenryu National Hospital; Masato Yamada, Department of Internal Medicine and Rheumatology, Juntendo University Shizuoka Hospital; Yasushi Ohno, Second Department of Internal Medicine, Gifu University Graduate School of Medicine; Yutaka Naito, Department of Neurology, Mie University Graduate School of Medicine; Yuji Tohda, Department of Respiratory Medicine and Allergy, Kinki University School of Medicine; Hiroo Yoshikawa, Division of Neurology and Stroke Care Unit, Department of Internal Medicine, Hyogo College of Medicine; Ryo Soda, Department of Internal Medicine, National Hospital Organization Minami-Okayama Medical Center; Nobuo Ueda, Department of Internal Medicine, Ehime Prefecture Central Hospital; Shunsuke Shoji, Allergy Section, National Hospital Organization Fukuoka National Hospital.
